# Selective deletion of forebrain glycogen synthase kinase 3β reveals a central role in serotonin-sensitive anxiety and social behaviour

**DOI:** 10.1098/rstb.2012.0094

**Published:** 2012-09-05

**Authors:** Camille Latapy, Véronique Rioux, Matthieu J. Guitton, Jean-Martin Beaulieu

**Affiliations:** 1 Department of Psychiatry and Neuroscience, Laval University, Quebec City, Quebec, Canada; 2 Faculty of Medicine, Department of Oto-Rhino-Laryngology and Ophthalmology, Laval University, Quebec City, Quebec, Canada; 3 Behavioural Phenotyping Core Facility, Mental Health University Institute of Quebec, Quebec City, Quebec, CanadaG1R 2G3

**Keywords:** serotonin, mood disorders, glycogen synthase kinase 3β, cortex, anxiety, sociability

## Abstract

Serotonin (5-HT) neurotransmission is thought to underlie mental illnesses, such as bipolar disorder, depression, autism and schizophrenia. Independent studies have indicated that 5-HT or drugs acting on 5-HT neurotransmission regulate the serine/threonine kinase glycogen synthase kinase 3β (GSK3β). Furthermore, GSK3β inhibition rescues behavioural abnormalities in 5-HT-deficient mice with a loss-of-function mutation equivalent to the human variant (R441H) of tryptophan hydroxylase 2. In an effort to define neuroanatomical correlates of GSK3β activity in the regulation of behaviour, we generated CamKIIcre-floxGSK3β mice in which the *gsk3b* gene is postnatally inactivated in forebrain pyramidal neurons. Behavioural characterization showed that suppression of GSK3β in these brain areas has anxiolytic and pro-social effects. However, while a global reduction of GSK2β expression reduced responsiveness to amphetamine and increased resilience to social defeat, these behavioural effects were not found in CamKIIcre-floxGSK3β mice. These findings demonstrate a dissociation of behavioural effects related to GSK3 inhibition, with forebrain GSK3β being involved in the regulation of anxiety and sociability while social preference, resilience and responsiveness to psychostimulants would involve a function of this kinase in subcortical areas such as the hippocampus and striatum.

## 1. Introduction

Neuropsychiatric disorders such as depression, bipolar disorders and schizophrenia represent a major public health problem, and a heavy burden for patients and their relatives [1,2]. The vast majority of pharmacological agents used for mental illnesses were discovered more than 50 years ago. However, while the primary molecular targets (e.g. receptors, monoamine transporters) of these drugs have been identified [3–6], the ultimate mechanisms responsible for their therapeutic actions remain poorly understood.

Monoamines—serotonin (5-HT), dopamine and norepinephrine—are the main neurotransmitters involved in the actions of most psychiatric pharmacological treatments [5,7,8]. An increase in extracellular 5-HT levels resulting from 5-HT transporter (SERT) blockade has historically been associated with the effects of antidepressants that are used for the treatment of major unipolar depression and other mood disorders [9–12]. Genetic evidence also supports a role for polymorphisms in genes encoding SERT or the rate-limiting enzyme for brain 5-HT synthesis [13,14], tryptophan hydroxylase 2 (TPH2), in depression [15–17]. However, the existence of such mutations does not appear to explain most cases of mood disorders in humans [18].

One approach to understand the role of 5-HT in the regulation of mood-related behaviour is to examine the functions of the signalling molecules that are regulated by the more than 15 identified 5-HT receptors [19]. Among these, the serine/threonine kinases glycogen synthase kinase 3 (GSK3) alpha and beta have been the targets of intensive research over the last decade [20,21]. These two iso-enzymes are the product of different genes termed *gsk3a* and *gsk3b*. Various drugs acting on 5-HT neurotransmission, such as selective serotonin reuptake inhibitors, monoamine oxidase inhibitors, tricyclic antidepressant and second-generation antipsychotics, are capable of inhibiting GSK3β activity by increasing the inhibitory phosphorylation of the N-terminal domain serine 9 [22–24]. Furthermore, the replacement of wild-type (WT) TPH2 by a R439H mutant form that is equivalent to the rare human R441H loss-of-function variant results in an approximately 80 per cent reduction of brain 5-HT synthesis, which was reported to be accompanied by an activation of cortical GSK3β in knockin mice [25]. The 5HT_1_ and 5HT_2_ receptor sub-types seem to play dualistic roles in regulating GSK3β activity [22,23]. In the brain of WT mice, GSK3β inactivation can be induced both by administration of the 5HT_1_ agonist 8-hydroxy-2-(di-n-propylamino)tetralin (8-OH-DPAT) or the 5HT_2_ antagonist, LY53857 [22]. However, administration of the 5HT_1_ antagonist WAY100635 or the 5HT_2_ agonist 2,5-dimethoxy-4-iodoamphetamine (DOI) does not affect the phosphorylation of GSK3β on the regulatory serine 9 residue [22]. The mechanisms through which 5HT_2_ antagonists regulate GSK3 are still unresolved, but most likely involve modulation of signalling by the G_α_q G protein to which these receptors are coupled. In contrast, the regulation of GSK3 by 5HT_1_ receptors appears to involve an activation of phosphatidylinositol 3-kinases (Pi3K) that in turn activates the serine/threonine kinase Akt, which then phosphorylates the inhibitory serine 9 residue of GSK3β [26].

In addition to its regulation by 5-HT, brain GSK3β is also inactivated by several neurotrophic factors, such as the brain-derived neurotrophic factor and its receptor TrkB through Pi3K-mediated signalling [27]. In contrast, activation of the dopamine D2 receptor (D2R) has been shown to activate GSK3 by triggering the formation of a signalling complex composed of Akt, beta-arrestin 2 and protein phosphatase 2A (PP2A) [28,29]. The formation of this complex leads to the inactivation of Akt by PP2A—and therefore relieves the inhibition of GSK3 by Akt [30]. N-methyl-d-aspartate (NMDA) receptor-dependent long-term potentiation (LTP) also activates GSK3β through a mechanism that seems to involve its dephosphorylation by protein phosphatase 1 [31].

Given the complexity and intricacy of the signalling pathways that can regulate brain GSK3 activity, it is highly probable that this kinase might contribute to the therapeutic effect of drug therapy on these different systems. Aside from 5-HT drugs, several psychoactive compounds can also modulate the activity of GSK3β *in vivo*. Among these, the psychostimulant amphetamine, which enhances dopamine tone, activates GSK3β following D2R stimulation [29,32,33]. In contrast, antipsychotic drugs with D2R antagonistic activity have an inhibitory effect on GSK3 [20,34]. Similarly, the NMDA receptor-blocker ketamine also inhibits GSK3β *in vivo*, an effect that can be associated with its action as a fast-acting antidepressant [35]. Finally, mood stabilizers lithium and valproic acid also trigger an inhibition of GSK3, either directly or by enhancing its inhibitory phosphorylation by Akt [36–40]. To establish the contribution of GSK3 to the effects of therapeutic agents, it is important to understand its roles in the regulation of behaviour by different neurotransmitter systems. In line with this, several reverse genetic and pharmacological approaches have allowed identification of key behaviours that can be affected by GSK3 activity *in vivo*.

Both the hyperactivity observed in mice lacking the dopamine transporter (DAT-KO mice) and hyperactivity induced by amphetamine administration can be reversed using pharmacologic inhibitors of GSK3 [32,41,42]. Increases in basal locomotion have also been reported in mice overexpressing GSK3β [43], whereas GSK3β haploinsufficient (GSK3β HET) mice [44] lacking 50 per cent of GSK3β protein expression display a significant reduction of behavioural responsiveness to amphetamine [32]. In contrast, mutant mice lacking the inhibitory phosphorylation sites on both GSK3 isoforms are hyper-responsive to amphetamine treatment [45]. Finally, the effects of lithium on novelty-driven exploratory locomotor behaviour appear to be dependent on the modulation of GSK3 activity [32,36].

Several data point towards a direct impact of GSK3 activity on behaviour in paradigms assessing emotional states [20,25,26,45]. Indeed, antidepressant-like responses to lithium or ketamine in tests measuring behavioural despair or anxiety have been shown to depend, at least in part, on the inhibition of GSK3 by these pharmacological agents [32,35,36]. In line with this, acute administration of pharmacological GSK3 inhibitors [36,46] provokes antidepressant-like responses in rodents in the tail suspension test (TST) and the forced swim test (FST). Similar changes in these tests have also been reported in GSK3β HET mice [25,47,48], whereas mutant mice lacking GSK3 inhibitory phosphorylation in both GSK3α and GSK3β isoforms display a general reduction of anxiety and depressive-like behaviours [45]. In addition, administration of the GSK3 inhibitor 4-benzyl-2-methyl-1,2,4-thiadiazolidine-3,5-dione (TDZD-8) or GSK3β haploinsufficiency rescue enhanced ‘anxiety’ and ‘behavioural despair’ phenotypes in 5-HT-deficient R439H tph2 knockin mice [25]. Finally, GSK3β can contribute to the regulation of social behaviour. Male homozygous R439H tph2 knockin mice display exacerbated aggression in a social interaction test, and that aggression is alleviated by GSK3β haploinsufficiency [25]. Furthermore, inhibition of GSK3 by lithium appears to restore social preference in mice lacking the fragile X syndrome gene *fmr1* [49]. However, the contribution of GSK3 to the regulation of sociability can be more complex since GSK3α knockout mice display a lack of social preference in the Crawley's sociability and preference for social novelty test [50,51], suggesting that the two GSK3 isoforms may play different roles in regulating social behaviours.

Taken together, these data present a complex picture of the roles of GSK3 in the regulation of behaviour. However, very little is known about the neuroanatomical determinants of these different effects and the possible contribution of 5-HT. Since cortical structures are believed to play a role in the regulation of mood, sociability and cognition by 5-HT [52–54], we undertook to examine the contribution of forebrain GSK3β in different mood-related behavioural paradigms known to be affected by the systemic activity of this kinase. To do so, we have generated a new animal model by breeding mice carrying deactivatable *gsk3b* floxed alleles with mice expressing the Cre recombinase postnatally in glutamatergic forebrain pyramidal neurons under the control of a Ca^2+^/calmodulin-dependent protein kinase II (CamKII) gene promoter.

## 2. Material and methods

### (a) Animals

The CamKIIcre-floxGSK3β line was obtained by breeding CamKIIcre mice [55] with a GSK3β flox line [56], following a standard two-step breeding protocol [57]. C57BL6J systemic GSK3β HET mice were described previously [44]. Because of the potential for recombination in sertoli cells of CamKIIcre-positive mice, all mice were obtained by breeding CamKIIcre-positive females (homozygous for floxGSK3β) with negative CamKIIcre males (homozygous for floxGSK3β). All mice tested were homozygous for GSK3β flox and either negative (NEG) or positives (POS) for the CamKIIcre transgene, leading to the inactivation of GSK3β postnatally in selected neuronal populations of POS mice. Because female CamKIIcre (POS)/GSK3β flox mice presented altered maternal behaviour, pups from this line were systematically raised by adoptive mothers. Male C57Bl6 and retired male breeder CD1 mice used as interaction partners for social behaviour tests were obtained from Charles River Laboratory (Senneville, Quebec, Canada).

All mice generated were housed by gender, in groups of two to five  per cage, under a 12 L : 12 D cycle, with ad libitum food and water. They were used at two to four months of age. Both male and female mice were used except for social tests, where only males were tested. Behavioural testing was performed between 8.00 and 13.00 (lights switched on at 7.00). Prior to all behavioural experiments, mice were housed in the experimental room at least 4 days before testing to allow for acclimation. Mice were left undisturbed in the room for 2 h before being killed for biochemical and tissue processing.

### (b) Antibodies

For Western blot analyses, GSK3 and actin were detected using mouse monoclonal IgG SC7291 (1 : 1000; Santa Cruz Biotechnology) and mouse monoclonal IgG MAB1501 (1 : 10 000; Millipore), respectively. The detection of GSK3 (S9/S21) phosphorylated form was performed using rabbit polyclonal IgG CST-9331 (1 : 500; Cell Signaling Technology). Secondary antibody IRDye 680 Goat Anti-Rabbit IgG LIC-926-32221 (1 : 10 000; Mandel) or IRDye 800CW Goat Anti-Mouse IgG LIC-926-32210 (1 : 10 000; Mandel) were then used.

For immunochemistry analysis, the following primary antibodies were used: rabbit polyclonal anti-GSK3β CST-9315 (1 : 500; Cell Signaling Technology), mouse monoclonal anti-neuronal nuclei (NeuN) MAB377 (1 : 250; Millipore), and mouse monoclonal anti-dopamine and cyclic AMP-regulated phosphoprotein (DARPP32) (BD611520; 1 : 1000; BD Transduction Laboratories). Revelation of labelling using the Odyssey imager (Licor Biotechnology, Lincoln, NE, USA) was performed using IRDye 680 Goat Anti-Rabbit IgG LIC-926-32221 (1 : 1000; Mandel) secondary antibody. Secondary antibodies used for confocal imaging were Alexa Fluor 488 goat anti-rabbit IgG and Alexa Fluor 568 goat anti-mouse IgG (1 : 1000; respectively, A-11008 and A-11004; Invitrogen, Burlington, ON, Canada).

### (c) Drug administration

Amphetamine (Tocris Bioscience, Ellisville, MO, USA) was prepared in a 0.9 per cent saline solution and injected (i.p.). TDZD-8 (EMD Biosciences, Inc., La Jolla, CA, USA) was injected i.p., after suspension in a minimal amount of Tween and bringing to volume with distilled water. TDZD-8 and vehicle solutions were administered 30 min prior to the TST (15 mg kg^–1^, i.p.).

### (d) Western blot analyses

Mice aged between two and three months were killed by decapitation, and their heads were immediately cooled by immersion in liquid nitrogen for a few seconds as described [32]. In each mouse, the striatum, prefrontal cortex, hippocampus and cerebellum were rapidly dissected (within 45 s) on an ice-cold surface, and frozen in liquid nitrogen before protein extraction. Tissue samples were homogenized in boiling 1 per cent sodium dodecyl sulfate (SDS) and boiled for 5 min. Protein concentration was measured using a DC-protein assay (Bio-Rad, Hercules, CA, USA). Protein extracts (25 µg) were separated on 10 per cent SDS/PAGE Tris-glycine gels (Invitrogen) and transferred to nitrocellulose membranes (Invitrogen). Blots were immunostained overnight at 4°C with primary antibodies. Immune complexes were revealed using appropriate IR-dye-labelled secondary antibodies. Quantitative analyses of fluorescent IR dye signal were carried out using an Odyssey Imager. For quantification, actin was used as a loading control for the evaluation of total protein levels, whereas respective total protein signals were used as loading controls for each phospho-protein signal. Results were further normalized to respective control conditions, in order to allow for comparison between separate experiments. The gels shown in the figures correspond to representative experiments, where each lane corresponds to a separate animal. Separate gels are presented within separate frames, and apparent signals may not be directly comparable between gel pictures.

### (e) Immunochemistry

Mice were deeply anaesthetised with ketamine-xylazine and perfused with 4 per cent paraformaldehyde (Sigma Aldrich, St. Louis, MO, USA). Brains were carefully removed and post-fixed overnight at 4°C. For Odyssey imaging, sagittal sections (45 µm) from adult mice were prepared using a vibratome (Leica Microsystems, Concord, Ontario, Canada), and permeabilized for two hours at room temperature with phosphate-buffered saline (PBS) + 0.5% Triton X-100 (Sigma Aldrich) + 3% bovine serum albumin (Sigma Aldrich) solution. The same solution was used to dilute primary and secondary antibodies. Brain slices were incubated overnight at 4°C with the primary antibodies, then washed and incubated with the secondary antibody for two hours at room temperature. After washing, slices were mounted with Prolong gold antifade reagent (Invitrogen), and scanned using the Odyssey imager at a resolution of 21 µm. For confocal imaging, the brains of five animals per genotype were incubated overnight in a PBS + 20% sucrose solution (Fisher Scientific, Ottawa, Ontario, Canada) after the post-fixation process. Three brains per genotype were used for evaluation of GSK3β expression in the cortex, hippocampus and striatum. Brains were embedded with Tissue-Tek Optimal Cutting Temperature compound (Pelco International, Redding, CA, USA) and rapidly frozen in cold 2-methylbutane (Sigma Aldrich). Sagittal sections (16 µm) from adult mice (aged two to three months) were prepared using a CM1900 cryostat (Leica Microsystems, Concord, Ontario, Canada). Remaining brains were used for evaluation of GSK3β expression in the amygdala (basomedial amygdaloid nucleus and lateral amygdaloid nucleus) and the nucleus accumbens (core). Coronal sections (45 µm) were prepared using a vibratome (Leica Microsystems, Concord, Ontario, Canada). The sections were labelled as already mentioned, and quantification was performed using a confocal microscope Axioskop 2 Mot plus (Carl Zeiss, Toronto, Ontario, Canada) equipped with argon 488 nm and helium-neon 543 nm lasers (LASOS Lasertechnik GmbH, Jena, Germany). Double-immunolabelled cells were analysed using Z-stack serial images and Axiovision V4.8.1.0 software.

### (f) Behavioural tests

#### (i) Locomotor activity

Locomotion was assessed under illuminated conditions in an automated Omnitech Digiscan apparatus (AccuScan Instrument, Columbus, OH, USA), as described [32]. For the evaluation of locomotor activity in a novel environment, mice were placed into the apparatus and their activity was monitored for 30 min. The following parameters were measured: horizontal activity, vertical activity and stereotypy count (repeated beam breaks). For tests involving drug treatment, mice were placed in the locomotor activity monitor chamber for an acclimation period of 1 h before being injected with amphetamine or vehicle. After injection, mice were returned to the monitoring chamber and their locomotor activity was recorded for an extra 2 h and 30 min.

#### (ii) Tail suspension test and forced swim test

Mice were tested using a tail suspension apparatus (Med-Associates, St. Albans, VT, USA), as described [58]. For the FST, mice were individually placed in a transparent glass cylinder containing clean water (kept at 25°C) to a height of 15 cm. In both tests, immobility time was computed for the total duration (6 min) and the last 4 min of the tests. In the TST, immobility time was extracted directly by the software. In the FST, the floating or immobility time (no limb movement and making only minimal movements to keep the head above water) was scored by two independent observers blind to the genotype.

#### (iii) Dark-light emergence test and open field

The dark-light box emergence test was performed for 5 min. Mice were initially placed in the centre of the dark chamber. The total number of transitions between chambers and the time spent in each side were automatically recorded. The open field test (OFT) was performed in an automated Omnitech Digiscan apparatus (AccuScan Instrument). Each mouse was placed in a corner of the box and the exploratory activity was recorded. Time spent in the centre, number of entries and distance travelled were recorded separately for the central (25% of the total surface) and peripheral areas.

#### (iv) Preference for social novelty

Male mice aged between two and four months were isolated for two weeks and placed in an apparatus designed according to Moy *et al*. [59]. They were allowed to circulate freely in the three-compartment box (60 × 40, 5 × 20 cm total) for 5 min. Empty inverted cups (Galaxy Pencil cup/Utility Cup; Spectrum Diversified Designs, Inc., Streetsboro, OH, USA) were placed in each outer compartment. After this acclimation session, experimental mice were placed in the central compartment with doors closed, allowing the experimenter to introduce an unknown male (age-matched, C57Bl6) in one of the cups, and an object of approximately the same size in the centre of the other inverted cup. Then, both doors were simultaneously opened and, for a 10 min session (session 1), the tested mouse was free to explore all three compartments. After this, the mouse was placed back in the centre compartment and the object was replaced by another unknown male mouse, while the first male remained in the same position. During the second session of the social novelty phase, the test mouse was again free to explore all three compartments for a 10 min session. During both test sessions, video tracking was performed, and the amount of time spent sniffing the stranger mice or the object was recorded and analysed using ANY-maze (Stoelting, Wood Dale, IL, USA).

#### (v) Social interactions

Social interactions were performed in a transparent Plexiglas area, with males being kept in isolation for 14 days before testing as described [60]. Unfamiliar pairs of age- and weight-matched animals were placed at the same time in the open field for 20 min. A control C57Bl6 male and a mouse from the tested line made up each pair. In each pair of animals, the following parameters were measured: time spent by the animals in active social interaction (including time spent sniffing, allogrooming, following, crawling, escaping and wrestling), and number of social (sniffing, following, allogrooming, crawling) or non-social events (grooming, escape, wrestling, biting) initiated by each animal.

#### (vi) Repeated social defeat

The defeat stress protocol was adapted from Berton *et al*. [61]. Experimental mice were exposed to a new aggressor (male CD1 retired breeder) each day for a 5 min period on 10 consecutive days. After 5 min of unrestricted contacts, the CD1 and tested mice remained in the same cage for 24 h, but were separated by a perforated Plexiglas cage-divider allowing for sensory contact, but preventing fights. Control animals were handled daily and housed in pairs in equivalent cages, but with members of the same strain. In order to assess the social avoidance, 6 days after the last defeat, control animals and defeated ones performed the first session of the preference for social novelty test, but in this case, an unfamiliar CD1 mouse was placed in the cup containing the social partner. Interaction time with the caged mouse was quantified as described previously.

#### (vii) Long-term social memory

In order to assess social memory, experimental male mice were housed with an unknown control C57Bl6 male (age- and weight-matched) in the same cage, but separated by a perforated Plexiglas cage-divider allowing for sensory contact. After 6 days of undisturbed housing, the C57Bl6 male and the cage-divider were removed, and the experimental mouse was housed for 2 days in the whole cage in order to reinforce social memory. Then, 3 days prior to the memory test, experimental mice were individually placed in new, clean cages. The assessment of social memory was done in the three-compartment box used in the preference for social novelty test. A first 5 min session was performed to habituate experimental mice to the apparatus and the empty inverted cups. Then, the ‘housemate’ of the experimental mouse and an unknown male were placed in each cup. The doors were opened and the experimental animal was free to explore the three-compartment box for 10 min. Video tracking was performed and the time spent sniffing the previous housemate or unknown mouse were determined.

### (g) Statistical analyses

Data from the biochemical and immunohistochemistry studies were analysed by two-tailed Student's *t*-tests. Behavioural studies were analysed using two-tailed *t*-tests or ANOVAs with Bonferroni *post-hoc* tests for multiple comparisons using GraphPad Prism software, v. 5.01 (Graphpad Software, La Jolla, CA, USA).

## 3. Results

### (a) Characterization of GSK3*β* expression in CamKIIcre-GSK3*β*flox mice

The CamKII-cre and flox GSK3β mice used to generate CamKIIcre-flox GSK3β mice have been thoroughly characterized previously [56,62]. Expression of the CamKII-cre transgene in this line is known to produce cre/flox-mediated recombination postnatally in pyramidal neurons of the forebrain [55] (the Jackson lab. http://cre.jax.org/Camk2a/Camk2a-creNano.html). In the absence of cre, the GSK3β floxed allele results in the production of a fully functional kinase and does not affect GSK3β gene expression, therefore allowing the use of cre-negative (NEG) mice as control animals for cre-positive (POS) ones.

**Figure 1.**
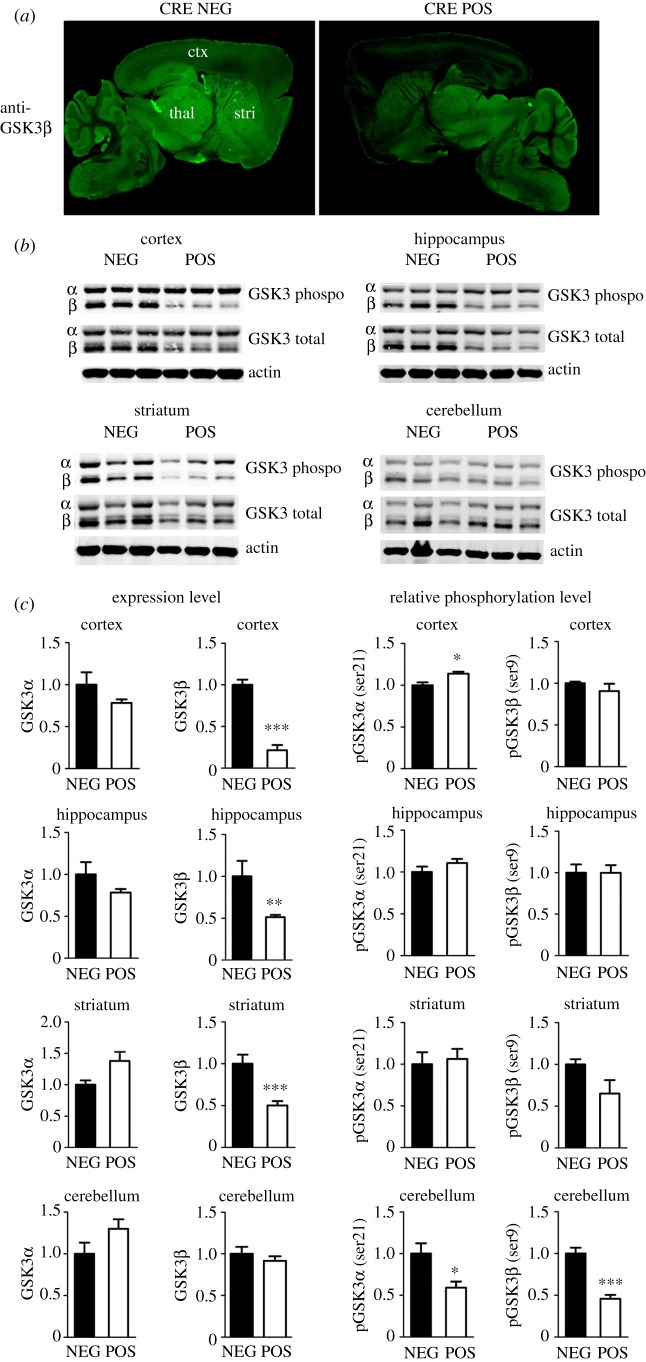
Pattern of GSK3β expression in CamKIIcre-GSK3βflox mice. (*a*) Sagittal brain sections (45 µm) of POS and NEG littermate mice reveal a tissue-specific deletion of GSK3β isoform in the cortex and hippocampus of POS mice compared with control animals. ctx, cortex; thal, thalamus; stri, striatum. (*b*) Representative Western blot analysis performed with brain extracts of NEG or POS animals for an evaluation of GSK3 expression and phosphorylation level. (*c*) Densitometric analysis of Western blots; *n* = 5 per condition. Data are presented as means ± s.e.m.; *p ≤ 0.05, **p ≤ 0.01, ***p ≤ 0.005, two-tailed Student's *t*-test.

To confirm the expression pattern of GSK3β in POS mice, immunostaining for GSK3β was performed on brain sections from adult POS mice and NEG littermates. The pattern of staining obtained reveals that, in POS mice, the intensity of GSK3β labelling was strongly reduced in the cortex and CA1 region of the hippocampus when compared with NEG littermates (figure 1*a*). This observation was confirmed by Western blot analyses for the total and phosphorylated (Ser9/Ser21) forms of both GSK3β and GSK3α. Results showed a significant reduction of GSK3β, but not of GSK3α expression, in tissue samples from the cortex, hippocampus and striatum, but no changes in the cerebellum of POS mice when compared with control NEG mice (figure 1*b*,*c*). Furthermore, deletion of GSK3β did not affect the inhibitory phosphorylation of GSK3α and residual GSK3β in most brain areas. However, a slight increase in GSK3α expression was found in the striatum, whereas deletion of neuronal forebrain GSK3β also resulted in limited reduction of the inhibitory phosphorylation of GSK3α and GSK3β in the cerebellum (figure 1*c*).

**Figure 2.**
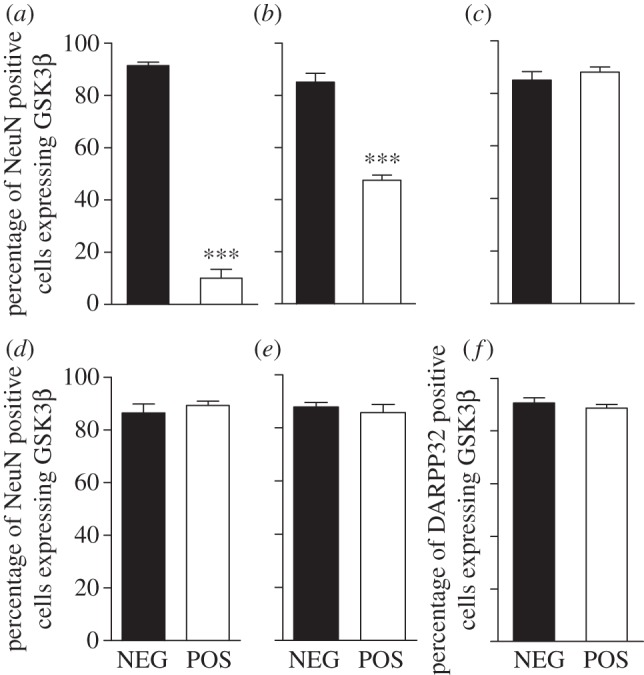
Loss of GSK3β expression in specific neuronal populations in CamKIIcre-GSK3βflox mice. Immunohistochemistry performed on sagittal brain sections (16 µm) of POS and NEG littermate mice reveals neuronal deletion of GSK3β in the cortex and CA1, but not in the amygdala and the striatum. (*a*) Percentage of cortical NeuN positive cells expressing GSK3β NEG (*n* = 402 cells) and POS (*n* = 407 cells). (*b*) Percentage of CA1 NeuN positive cells expressing GSK3β NEG (*n* = 417 cells) and POS (*n* = 432 cells). (*c*) Percentage of CA3 NeuN positive cells expressing GSK3β NEG (*n* = 395 cells) and POS (*n* = 336 cells). (*d*) Percentage of amygdala NeuN positive cells expressing GSK3β NEG (*n* = 395 cells) and POS (*n* = 412 cells). (*e*) Percentage of nucleus accumbens (NAc) NeuN positive cells expressing GSK3β NEG (*n* = 453 cells) and POS (*n* = 487 cells). (*f*) Percentage of striatal DARPP32 positive cells expressing GSK3β NEG (*n* = 213 cells) and POS (*n* = 221 cells). Data are presented as means ± s.e.m.; ***p ≤ 0.005, two-tailed Student's *t*-test.

We used more specific immunohistological characterization of GSK3β expression in POS mice to quantify the loss of GSK3β at a cellular level. First, a co-labelling of GSK3β protein and NeuN was used to determine the number of mature neurons expressing GSK3β in the cortex, hippocampus, amygdala and nucleus accumbens (NAc). The percentage of NeuN positive cells expressing GSK3β was reduced by approximately 90 per cent in the cortex of POS mice (figure 2*a*), whereas this reduction was only approximately 45 per cent in the CA1 region of the hippocampus (figure 2*b*), and remained unchanged in CA3 (figure 2*c*) as well as in the amygdala (figure 2*d*). The ratio of striatal NeuN positive cells in the NAc and DARPP32 positive medium spiny neurons expressing GSK3β in the striatum was also unchanged between NEG and POS mice (figure 2*e*,*f*). This indicates that reduction of striatal GSK3β levels obtained in Western blot analysis most probably results from a lack of GSK3β in cortico-striatal projections and not from a reduction of GSK3β expression in striatal neurons.

### (b) Effects of forebrain GSK3*β* inactivation on dopamine-mediated locomotion

**Figure 3.**
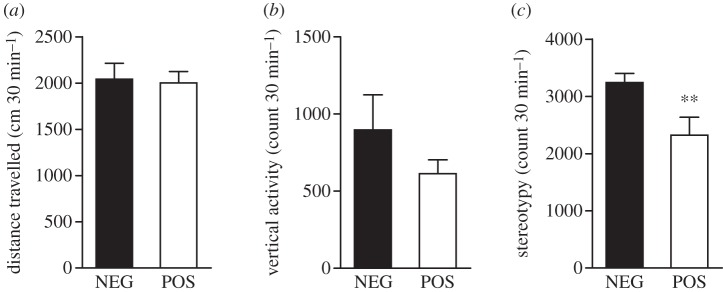
The effect of forebrain GSK3β inactivation on novelty-driven locomotor behaviour. (*a*) 30 min locomotor activity in a novel environment for NEG (*n* = 17) and POS (*n* = 14) mice. (*b*) 30 min vertical activity in a novel environment for NEG (*n* = 17) and POS (*n* = 14) mice. (*c*) 30 min stereotypy count in a novel environment for NEG (*n* = 17) and POS (*n* = 14) mice. Data are presented as means ± s.e.m.; **p ≤ 0.01, two-tailed Student's *t*-test.

Mice expressing a constitutive active form of GSK3β develop mild hyperactivity in a novel environment [45], which is reminiscent of the behaviour of hyperdopaminergic DAT-KO mice [63]. Furthermore, inhibition of GSK3β antagonizes dopamine-mediated hyperactivity in normal mice treated with amphetamine as well as in DAT-KO mice [28,32]. When placed in a locomotor activity monitor for a period of 30 min, CamKIIcre-floxGSK3β POS mice did not show any difference in basal horizontal or vertical activity when compared with their NEG littermates (figure 3*a*,*b*). However, they exhibited a slight reduction in stereotypy, as measured in the number of repeated beam breaks over the duration of the test (figure 3*c*).

**Figure 4.**
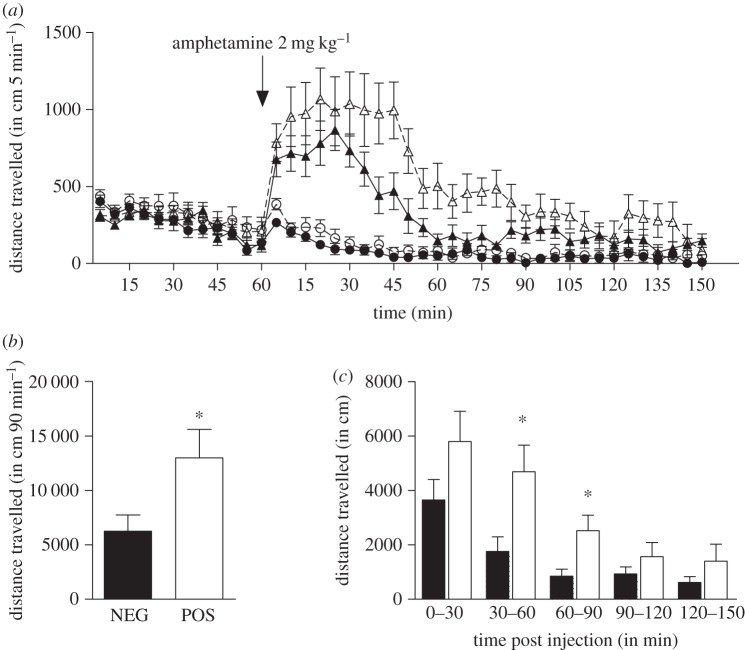
Response to acute amphetamine treatment. (*a*) Time course of locomotor-induced behaviour in response to an i.p. injection of 2 mg kg^–1^ amphetamine or saline solution in NEG (filled circles, vehicle (*n* = 8); filled triangles, amphetamine 2 mg kg^–1^ (*n* = 8)) and POS (unfilled circles, vehicle (*n* = 8); unfilled triangles, amphetamine 2 mg kg^–1^ (*n* = 7)) mice, expressed in distance travelled per 5 min blocks. (*b*) Cumulated distance travelled during the first 90 min after amphetamine treatment in NEG (*n* = 8) and POS (*n* = 7) mice. (*c*) Time course response to amphetamine of NEG (filled bars, *n* = 8) and POS (unfilled bars, *n* = 7) mice expressed in distance travelled per 30 min blocks. Data are presented as means ± s.e.m.; *p ≤ 0.05. Two-tailed Student's *t*-test was used.

To further explore the role of GSK3β in the behavioural effects of amphetamine, an effective dose (2 mg kg^–1^ of body weight i.p.) was administered to POS and NEG animals. Amphetamine induced hyperlocomotion in NEG animals as well in POS mice (figure 4*a*). Quantification of locomotor activity for a period of 90 min following drug administration revealed an overall stronger responsiveness to amphetamine in mice with forebrain GSK3β deficiency (figure 4*b*). A time-course analysis of the whole duration of the test showed that, whereas POS and NEG mice have similar responses to amphetamine initially, POS mice were significantly more responsive to amphetamine, between 30 and 90 min after drug administration (figure 4*c*). These observations are in stark contrast to the overall reduction in locomotor responsiveness to amphetamine reported in systemic GSK3β HET mice [32], and suggests that GSK3β expressed in different brain regions may play differential roles in modulating responsiveness to this drug.

### (c) Involvement of forebrain GSK3*β* in the regulation of anxiety

Inhibition of GSK3β has been shown to replicate several effects of antidepressants in tests measuring behavioural despair or anxiety-like behaviours in rodents [25,46,47,64]. We investigated the effects of a selective GSK3β deficiency in two sets of tests used to measure antidepressant-like effects. A first group of tests, made up of the Porsolt FST and the TST, was used to assess antidepressant effects on behavioural despair. A second group of tests, including the OFT and the dark-light emergence test (DLET), measured the effects of drugs on anxiety-related behaviours. Notably, we have previously shown that systemic inhibition of GSK3β reverses the behavioural effects of 5-HT deficiency resulting from the expression of mutant R439H-tph2 in the DLET and the TST [25].

**Figure 5.**
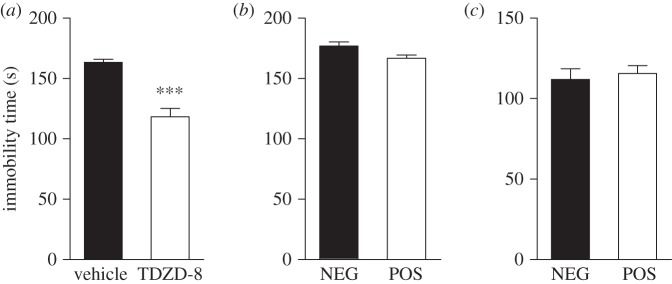
Absence of antidepressant-like response in tests of ‘behavioural despair’ in mice lacking forebrain GSK3β. (*a*) Immobility time in the tail suspension test (TST; 4 min) in C57 WT in response to an i.p. injection of 15 mg kg^–1^ of TDZD-8 (*n* = 7) or vehicle solution (*n* = 7). (*b*) Immobility time in the TST (4 min) in NEG (*n* = 13) and POS (*n* = 12) mice. (*c*) Immobility time in the forced swim test (4 min) in NEG (*n* = 16) and POS (*n* = 10) mice. Data are presented as means ± s.e.m. ***p ≤ 0.005. Two-tailed Student's *t*-test was used.

Systemic inhibition of GSK3β using inhibitors or genetic approaches can mimic the effects of antidepressants and lithium by increasing the time spent by animals struggling against an inescapable situation (figure 5*a*) [46,64]. By convention, this is indicated by a reduction in the animal's immobility time. When assessed in these two tests, cre-positive GSK3β flox mice did not differ from their cre-negative littermates in immobility time, as measured over the last 4 min (figure 5*b*,*c*) or over the whole duration of the test (data not shown). This suggests that neither cortical nor hippocampal CA1 GSK3β is responsible for the behavioural response to GSK3β inhibition.

**Figure 6.**
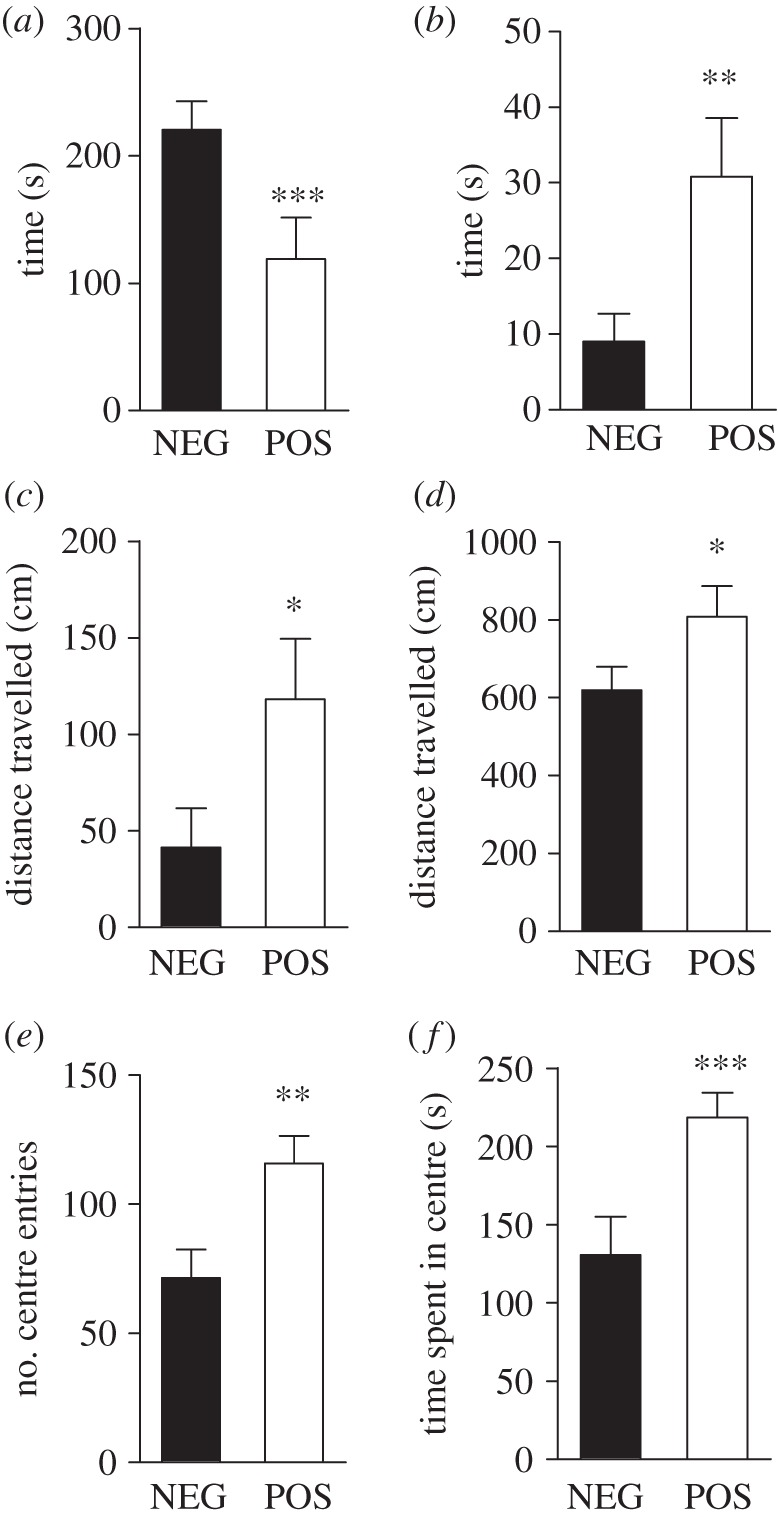
Deficit in GSK3β forebrain expression elicits anxiolytic responses. (*a–d*) Dark-light emergence test in NEG (*n* = 13) and POS (*n* = 12) mice. (*a*) Latency to cross to the light chamber. (*b*) Time spent in the light chamber. (*c*) Activity in light chamber. (*d*) Total distance travelled in the dark-light emergence box. (*e*,*f*) Open field in NEG (*n* = 14) and POS (*n* = 7) mice. (*e*) Number of centre entries. (*f*) Time spent in the centre. Data are means ± s.e.m.; *p ≤ 0.05, **p ≤ 0.01, ***p ≤ 0.005. Two-tailed Student's *t*-test was used.

In contrast, results obtained using the OFT and the DLET revealed a marked reduction in the basal level of anxiety-related responses in POS mice when compared with control NEG littermates (figure 6). In DLET, reduction in anxiety levels was detected by measuring rodents’ decreased avoidance of an ‘anxiogenic’ brightly illuminated compartment that is opposed to a ‘non-anxiogenic’ dark compartment. When exposed to the DLET, POS mice presented a markedly reduced latency to cross to the illuminated chamber (figure 6*a*), spent more time and were more active in this compartment (figure 6*b*,*c*), resulting in an overall increase in total activity (figure 6*d*). In the OFT, reduced anxiety is characterized by a reduction in the avoidance of the ‘anxiogenic’ centre of an illuminated open field. In this test, POS mice displayed a higher number of centre entries and an augmentation of the time spent in the central area. Overall, results obtained using the DLET and OFT in mice with forebrain GSK3β deficiency are consistent with results obtained following a systemic inhibition of GSK3β [25,36], indicating a probable contribution of cortex and/or hippocampus CA1 GSK3β in the regulation of anxiety-related behaviours.

### (d) Involvement of forebrain GSK3*β* in social behaviour

In addition to the action on anxiety and depressive-like behaviour, the reduction of GSK3β activity also antagonizes the effects of a reduced 5-HT synthesis on social behaviour and aggression in mice expressing mutant TPH2 [25]. In order to assess the involvement of forebrain GSK3β in social behaviour, male CamKIIcre-GSK3βflox mice were assessed in two different tests, the preference for social novelty test and a social interaction test. Because there are few studies on the role of GSK3β in regulating social behaviour, systemic GSK3β HET mice were also used as a contrast group in these tests. We are aware that GSK3β HET and CamKIIcre-GSK3βflox could not be compared directly, owing to differences in genetic backgrounds and level of local GSK3β deficiency [48,65]. However, a critical side-by-side comparison of the behaviour between these and CamKIIcre-GSK3βflox mice can nevertheless provide indications about the possible contribution of non-forebrain GSK3β in the regulation of social behaviour.

**Figure 7.**
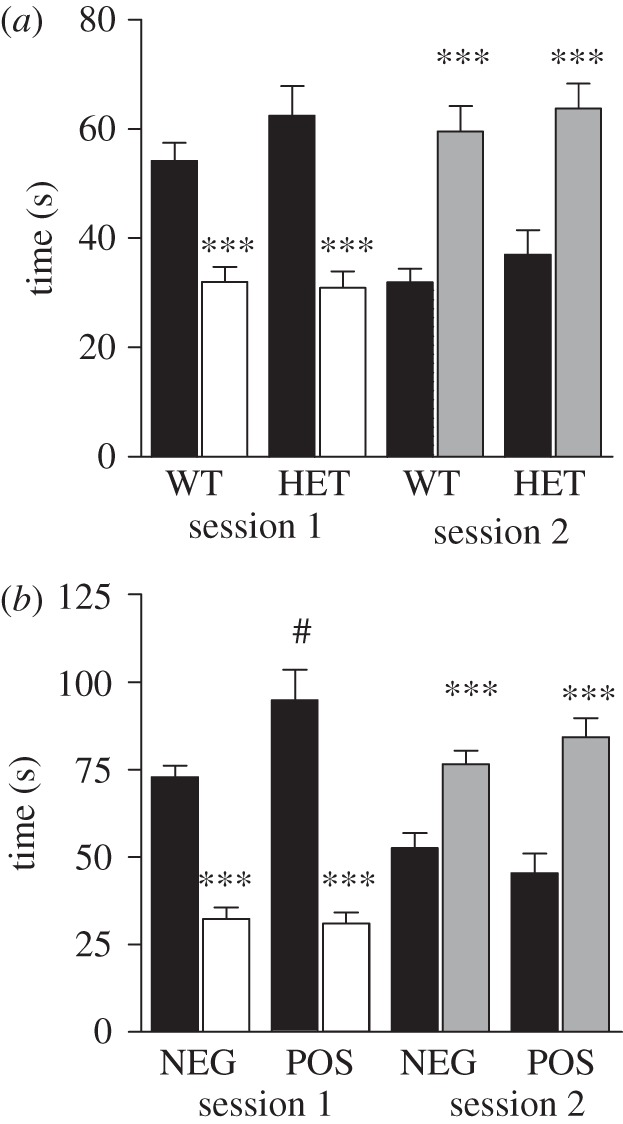
The effect of GSK3β expression on social preference and recognition. (*a*) Average time spent sniffing a C57WT stranger mouse or an object during the place preference test for the GSK3β HET mice. WT: *n* = 10; HET: *n* = 10. (*b*) Average time spent sniffing a C57WT stranger mouse or an object during the place preference test for the forebrain GSK3β-deleted mice. NEG: *n* = 10; POS: *n* = 10. Data are means ± s.e.m.; # *p* ≤ 0.05, ***p ≤ 0.005. One-way ANOVA followed by a Bonferroni post-hoc test. Asterisks represent statistical preference during a same session; hash represents statistical difference between genotypes. Black bars, stranger 1; white bars, object; grey bars, stranger 2.

The social preference test is designed to measure mice's preferences for social interactions and their capacity to distinguish and prefer a new social partner over a partner that is already known. In the first part of the test, the animal is placed in the centre of a three-compartment box and may choose between two adjoining compartments containing either a caged novel social interaction partner or a caged novel object. In the second part of the test, the mouse is given the choice between a known interaction partner and a novel one. Interestingly, inhibition of GSK3 by lithium has been reported to restore social preference in mice lacking the fragile X syndrome-associated gene *fmr1*, but not in WT control mice [49]. Furthermore, mice lacking the GSK3α isoform display an absence of social preference in this test [50]. When assayed in the social preference tests, GSK3β HET and cre-positive GSK3βflox mice did not exhibit overt deficits (figure 7). As may be expected [51,59], during the first session of the preference for social novelty test, mice of all genotypes spent more time with the unknown partner than with the object (figure 7). Similarly, GSK3β HET mice and cre-positive GSK3βflox mice and their respective control littermates also showed a comparable preference for the new interaction partner over the old one in the second portion of the tests. However, in contrast to their NEG littermates, POS mice spent significantly more time sniffing the first interaction partner than did their NEG littermates (figure 7*b*). This slightly higher interaction with an unknown mouse, as opposed to an object, was observed only in mice with forebrain GSK3β depletion. This observation suggests that social interest may be higher in these mice. However, even if the social preference test gives an evaluation of sociability and social memory, it is important to note that, in this case, the tested mouse is free to explore the entire apparatus, whereas the interacting partners are always encaged. On the one hand, this situation does not fully allow reciprocal social behaviours. On the other hand, the sniffing time of tested mice can be affected by different stress signals emitted by the caged interaction partner.

**Figure 8.**
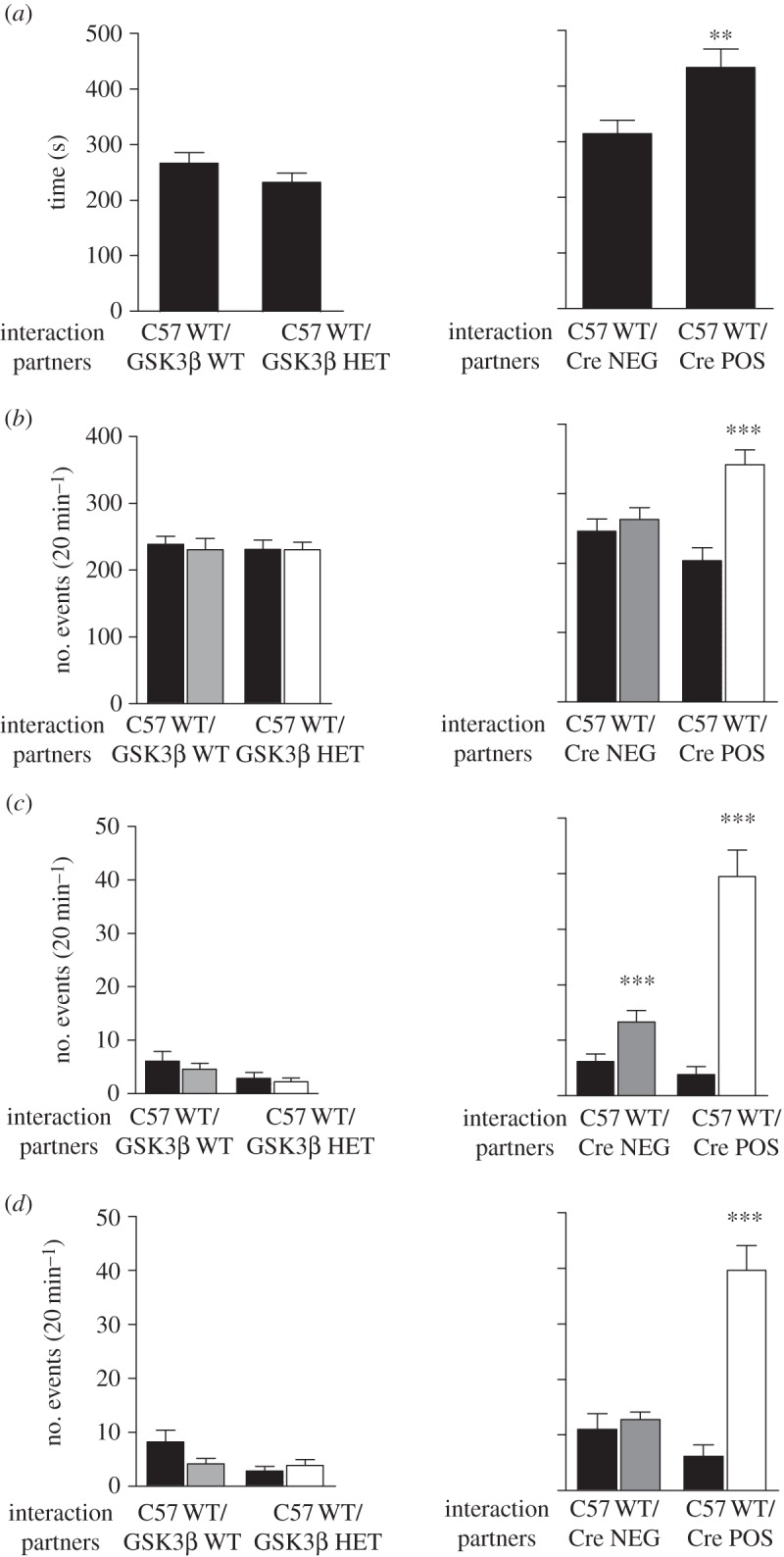
Social interaction test in an open area. (*a*) Time (in sec) spent in active social interaction during a 20 min period. (*b*) Number of social events initiated by each of the interacting mice during the entire testing session. (*c*) Number of following events initiated by each of the animals during the entire testing session. (*d*) Number of allogroomings events initiated by each of the animals during the entire testing session. Pairs of animals consisted of C57 WT/GSK3β WT mice (*n* = 10), C57 WT/GSK3β WT mice (*n* = 10), C57 WT/NEG mice (*n* = 15), C57 WT/POS mice (*n* = 10). Data are presented as means ± s.e.m.; **p ≤ 0.01, ***p ≤ 0.005. One way ANOVA followed by a Bonferroni post-hoc test. (*b*–*d*) Black bars, C57 WT; grey bars, GSK3β WT (left panels) or Cre NEG (right panels); white bars, GSK3β HET (left panels) or Cre POS (right panels).

In an effort to evaluate sociability under more ecological conditions, a social interaction test was performed. In this case, both tested mice and their interaction partners were free to explore the environment and interact with each other. Active social interaction time, made up by time during which two unfamiliar interacting mice are engaged in social activities within an open area, was measured. We observed no difference between GSK3β HET and their WT littermates in this test (figure 8). In contrast, the total active social interaction time was higher in POS mice than in NEG mice (figure 8*a*). More specifically, the number of social events initiated by POS mice was elevated (figure 8*b*), and the number of specific types of social events such as followings and allogroomings was particularly increased (see the figure 8*c*,*d*, and electronic supplementary material, movies S1 and S2). Taken together, these data support the idea of a predominant role of forebrain GSK3β activity in the regulation of social behaviours.

**Figure 9.**
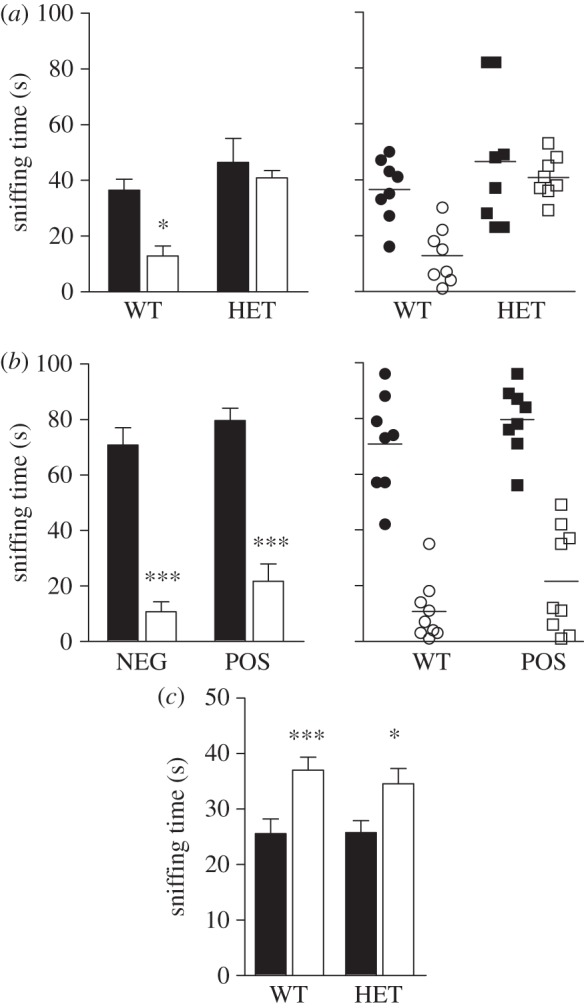
Differential effects of global and forebrain GSK3β depletion on social interaction behaviour induced by chronic social defeat stress. (*a*) Group (left) and individual (right) representations of time spent sniffing a CD1 stranger mouse after the repeated social defeat stress in GSK3β HET mice. Black bars, squares and dots represent control animals not exposed to repeated social defeat stress (WT: *n* = 8; HET: *n* = 8). White bars, squares and dots represent socially stressed mice (WT: *n* = 9; HET: *n* = 9). (*b*) Group (left) and individual (right) representations of time spent sniffing a CD1 stranger mouse after the repeated social defeat stress in POS mice. Black bars, squares and dots represent control animals not exposed to repeated social defeat stress (NEG: *n* = 8; POS: *n* = 8). White bars, squares and dots represent socially stressed mice (NEG: *n* = 9; POS: *n* = 9). (*c*) Assessment of social memory in GSK3β haploinsufficient mice and their WT littermates (WT: *n* = 10; HET: *n* = 9). Filled bars, familiar mouse; unfilled bars, unfamiliar mouse. Data are presented as means ± s.e.m.; *p ≤ 0.05, ***p ≤ 0.005. In (*a*) and (*b*), one-way ANOVA with Bonferroni post-hoc test was used. In (*c*), two-tailed Student's *t*-test was used.

Given the effects observed on anxiety and social behaviour, we then explored whether forebrain GSK3β is also involved in resilience to social stress. Recent evidence suggested a role for GSK3β in regulating resilience in a social defeat model of depression [66]. A repeated social defeat test was performed, followed by measurement of the social avoidance that it induced. In WT GSK3β mice, the repeated social defeat protocol induced a reduction of interest for an unknown mouse, characterized by social avoidance (figure 9*a*). A similar response to repeated social defeat was also observed in both POS and NEG GSK3βflox (figure 9*b*). Interestingly, GSK3β HET mice did not develop social aversion after chronic social defeat (figure 9*a*), thus supporting the contribution of GSK3β inhibition in resilience. Furthermore, even if GSK3β HET mice show poor memory reconsolidation [67], social memory does not seem to be affected (figure 9*c*), allowing us to exclude the hypothesis relating the absence of social avoidance to a lack of long-term social memory. This indicates that global GSK3β haploinsufficiency is sufficient to prevent social aversion, as induced by the repeated social defeat test, and that forebrain GSK3β is not a major factor explaining the lack of social avoidance observed in GSK3β HET mice.

## 4. Discussion

Modulation of 5-HT neurotransmission either by antidepressants or second-generation antipsychotics is central to psychopharmacology [4,6,10,68]. Among different signalling molecules that can be regulated by 5-HT, several lines of evidence support a role for GSK3 in signalling networks underlying the development and treatment of mental illnesses [21,26,69]. In line with this, systemic inhibition of GSK3β has been shown to have effects similar to those of mood stabilizers, antipsychotics or antidepressants in several behavioural tests used to model endophenotypes of psychiatric disorders in rodents. Several psychiatric drugs (e.g. lithium, clozapine, fluoxetine and ketamine) have been demonstrated to inhibit GSK3 *in vivo* [20]. Interestingly, drugs from these different categories are often prescribed jointly as part of combination therapies. However, different types of therapeutic agents still have very different clinical efficacies in treating distinct mental illnesses [19]. This suggests that inhibition of GSK3 may be a core mechanism of action for different classes of psychiatric drugs, but that modulation of other signalling pathways may be responsible for their clinical differences [19]. Alternatively, most of these drugs bind directly to specific cell-surface proteins that are expressed by restricted neuronal populations [6,70]. Therefore, various classes of pharmacological compounds may differentially modulate GSK3 activity in distinctive neuronal networks.

In order to understand the specific contributions of GSK3 inhibition in the effects of psychiatric drugs, it is important to map the roles played by these ubiquitous protein kinases in the modulation of behaviour to different brain areas. As a first step towards this objective, we have generated conditional knockout mice lacking GSK3β postnatally in pyramidal forebrain neurons. Characterization of protein expression in these mice confirmed that the expression of GSK3β is supressed in most pyramidal neurons of the cortex and in approximately 50 per cent of neurons of the hippocampal CA1 region. Furthermore, regional suppression of GSK3β activity did not overly affect the expression of brain GSK3α. Therefore, it is highly probable that most behavioural modifications observed in POS CamKIIcre-GSK3βflox mice result from a major contribution of cortical GSK3β. However, a limitation of this animal model is that contributions of CA1 pyramidal neurons from the hippocampus cannot be excluded. More delimited GSK3β deletion using transgenic mice or viral vectors expressing cre in a more restricted pattern will be needed to further refine the functional neuroanatomical mapping of GSK3β activity in neuronal functions that we have initiated here.

That being said, results from the behavioural characterization of CamKIIcre-GSK3βflox mice clearly indicate major differences in the consequences of cortical and subcortical GSK3β inhibition on a variety of behavioural responses associated with mood regulation. Among effects that can be associated with systemic inhibition of GSK3, three were not affected or affected in a different way in mice with forebrain GSK3β deficiency. Locomotor response to amphetamine is reduced both in GSK3β HET and in mice treated with GSK3 inhibitors [32,45,65,71]. In contrast, no reduction of locomotor response to amphetamine was noted in cre-positive GSK3βflox mice, therefore suggesting that this effect of GSK3β inhibition is mediated by subcortical structures. Considering that amphetamine exert its effects by triggering dopamine release [72], the most probable neuronal type responsible for the inhibition of amphetamine responses by GSK3 inhibitors would be medium spiny neurons of the striatum, in which activation of D2 dopamine receptors in response to amphetamine activates GSK3β [29]. Further studies using more specific gene promoters such as D1 and D2 dopamine receptors to drive cre expression in specific subtypes of medium spiny neurons [73] should allow definite confirmation of this hypothesis in the future.

Systemic inhibition of GSK3β has also been shown to exert antidepressant-like effects in the TST (figure 5*a*) and the FST [25,36,46–48,64,71]. Interestingly, these effects were absent in cre-positive GSK3βflox mice. This observation is concordant with recent data showing an antidepressant-like effect of lentiviral vectors expressing siRNA targeted against GSK3β transcripts in the hippocampal dentate gyrus of stressed mice using these same behavioural paradigms [74]. Therefore, it seems possible that the overall antidepressant-like effects of systemic GSK3β inhibition can be explained, at least in part, by an inhibition of this kinase in the dentate gyrus. However, further studies examining the possible contribution of other brain areas will be needed to more firmly establish this possibility.

Examination of the role of brain GSK3β in social preference indicates that, under normal conditions, partial systemic inhibition of GSK3β or a more pronounced reduction of GSK3β expression in forebrain neurons does not affect social preference or the recognition and preference of a novel interaction partner. This is in contrast with data obtained in GSK3α knockout mice, which showed a lack of social preference in this same test [50] and further support the possibility that both isoforms of GSK3 may differently contribute to social preference. In contrast, examination of the contribution of GSK3β in the behavioural response to repeated social defeat stress showed, for the first time, that a global reduction of GSK3β expression is sufficient to confer resilience. However, this effect appears to be dependent on subcortical structures, because mice with cortical GSK3β deficiency were not resilient in this same test. Interestingly, an activation of GSK3β in the nucleus accumbens has recently been reported to correlate with the development of social defeat in susceptible mice [66]. Moreover, expression of a dominant negative form of GSK3 in this same brain region increased resilience. This is concordant with our observations (figure 9) and suggests that a modulation of GSK3β activity in subcortical structures such as the nucleus accumbens may play a major role in mediating resilience to social stress, at least in this specific rodent model.

The overall effect of forebrain GSK3β depletion is a reduction in anxiety that is combined with an increase in the initiation of social interaction. Interestingly, this pattern of behavioural response can be compatible with a modulation of 5-HT_1A_ receptor signalling. Previous studies have shown that GSK3β would be mostly activated by 5-HT_2A_ receptors and inhibited by 5-HT_1A_ receptors [22,23,26]. Therefore, the removal of GSK3β expression in the forebrain in POS CamKIIcre-GSK3βflox mice may partly mimic the effects of an activation of 5-HT_1A_ receptor. Activation of this receptor by specific agonists has anxiolytic effects in mice and humans [75,76], whereas inactivation of 5-HT_1A_ receptors in mice is anxiogenic [77–79]. Similarly, modulation of the activity of this receptor in the prefrontal cortex has also been associated with the regulation of social behaviour and aggression [80,81].

Alternatively, behavioural changes induced by forebrain GSK3β inhibition may also result from changes in responsiveness to glutamatergic neurotransmission. Indeed, activation of GSK3β in cortical neurons antagonizes glutamatergic responses by reducing NMDA-receptor cell surface expression [82]. Similarly, GSK3 activity has also been shown to be essential for reduced glutamate neurotransmission during hippocampal long-term depression [31,83]. While these explanations remain speculative, they fit relatively well with our behavioural observations in POS CamKIIcre-GSK3βflox mice, but further detailed investigations will be needed to clarify this issue.

Overall, changes in the regulation of GSK3β activity have been associated with the actions of several psychoactive drugs, including those affecting 5-HT functions in the treatment of mood disorders. Our characterization of mice lacking this kinase in forebrain pyramidal neurons has shown that its contribution to different mood-associated behaviour is highly neuroanatomically defined. A better understanding of the functions of GSK3β in different brain areas may be the key to unravel the mechanisms by which it contributes to the regulation of mood.
